# Individualized approach to palliative care integration for patients with metastatic non-small cell lung cancer

**DOI:** 10.3389/fmed.2026.1921610

**Published:** 2026-09-02

**Authors:** Carley Mitchell, Roshni S. Kailar, Melinda L. Hsu

**Affiliations:** 1 Department of Hematology and Oncology, University Hospitals Seidman Cancer Center, Cleveland, OH, United States; 2 Case Western Reserve University School of Medicine, Cleveland, OH, United States; 3 Department of Medicine, University Hospitals Cleveland Medical Center, Cleveland, OH, United States; 4 University Hospitals Seidman Cancer Center, Cleveland, OH, United States

**Keywords:** cancer, lung cancer, pallitative care, precision, precision medicine

## Abstract

Early integration of palliative care is recommended for all patients with advanced solid tumor malignancies. However, this practice has not been widely adopted for various reasons. Recent therapeutic advancements along with improved understanding of tumor biology have contributed to greater prognostic uncertainty and changes in symptom burden, highlighted nicely within the metastatic non-small cell lung cancer population. These changes call for a more nuanced approach to palliative care referral which could be facilitated through patient reported outcome monitoring and disease related triggers.

## Introduction

Palliative care is an evolving medical subspecialty focused on relieving suffering and improving quality of life through symptom management along with psychosocial and spiritual support for patients with advanced illnesses (1, 2). This can be incorporated into patient care at any point in the disease trajectory. Accomplishing this requires a collaborative approach among physicians, nurses, social workers, and chaplains among others to meet individual patient needs (2). While palliative care is utilized among a wide range of diseases, cancer diagnoses are a leading driver of palliative care referrals due to both high symptom burden and poor prognosis. Therefore, oncology and palliative care providers often work closely together providing care for mutual patients. In fact, current recommendations from the American Society of Clinical Oncology urge early integration of palliative care for all patients with advanced solid tumor malignancies (3). This recommendation comes from a breadth of work within the cancer population, much of which includes or focuses on those with metastatic non-small cell lung cancer (NSCLC). This is primarily due to the fact that this disease is both highly debilitating and has historically held a limited prognosis, often cited as less than one year (4). However, in recent decades cancer therapeutics have evolved becoming more precision medicine focused with advances in metastatic NSCLC leading the way. In concert with this changing therapeutic landscape, a more nuanced approach to palliative care integration is warranted. Here we will discuss the historic context for early palliative care referral, challenges in widespread adoption of this practice and ways to implement precision palliative care.

## Historic context of early palliative care integration

Several studies have highlighted the importance of early palliative care integration through superior survival and patient reported outcomes. When considering survival, the seminal randomized controlled trial published in 2010 by Temel and colleagues is frequently cited. Here, patients with metastatic NSCLC were randomized to receive either early palliative care plus standard oncologic care or standard oncologic care alone. Early palliative care consisted of monthly palliative care visits with the first visit occurring within three weeks of enrollment or eight weeks of diagnosis while standard oncologic care incorporated palliative care only when requested by the patient, family or oncologist. Those that received early palliative care experienced a longer median survival by 2.7 months (4). Later, the ENABLE III trial supported a 1-year survival advantage of 63% vs. 48% for patients with advanced cancer who received an early palliative care intervention, within 30–60 days of diagnosis, as opposed to the same intervention being delayed at least three months following diagnosis (5). While this study encompassed multiple advanced cancer diagnoses, those with lung cancer accounted for nearly 43% of the entire cohort (5). Not only is the timing of palliative care involvement important, but the frequency of care may also augment outcomes. In a South Korean cohort of patients with advanced solid tumor malignancies, with lung cancer comprising near 13%, and an estimated life expectancy of 12 months or less, patients were randomized to receive either early palliative care alongside usual oncologic care or usual oncologic care alone. The palliative care intervention included both outpatient consultations and telephone coaching. Here, the probability of survival at 2 years was significantly increased in a dose dependent fashion if the palliative care intervention was received 10 or more times (6). Overall, the mechanism by which palliative care integration leads to improved survival outcomes is likely multifactorial. Symptom control and psychosocial support decreases levels of distress which has been shown to positively impact survival. Further, treatment adherence is improved when the above factors are optimized (7). Finally, palliative care is associated with less aggressive treatment near end-of-life thereby decreasing exposure to potentially harmful toxicity which may inadvertently hasten death (7).

While survival is an important outcome measure for both patients and providers, the additional effect of palliative care on quality of life and symptom burden should not be understated. A 2017 Cochrane review assessing early palliative care for adults with advanced cancer incorporated 7 unique trials. While the certainty of evidence was considered low due to risk of bias, they reported a significant improvement in health-related quality of life for those receiving early palliative care as evidenced by a standard mean difference (SMD) of 0.27 (95% CI 0.15–0.38), or a 4.59-point improvement in the Functional Assessment of Cancer Therapy (FACT) - General assessment, which evaluates physical, social, emotional and functional wellbeing, among a general cancer population (7). Additionally, symptom intensity was significantly lower in the setting of early palliative care with a SMD of −0.23 (95% CI −0.35–−0.10) correlating to an average 35.4-point reduction on the Edmonton Symptom Assessment Scale (ESAS) (7). These results coincide with clinically meaningful change (7). Since publication of this Cochrane review, additional research has been published and incorporated into more recent meta-analyses including one conducted by Huo et al. In this publication, 16 trials were included revealing a more robust improvement in quality of life with a pooled SMD of 0.737 (95% CI 0.240–1.1234) with additional significant advantages in both symptom burden and mood (8).

Despite the clear benefits and guideline recommendations for early palliative care, several challenges exist preventing broad-based integration. This may include inadequate knowledge from both patients and providers regarding the benefits and scope of palliative care along with socioeconomic factors that may limit a patient's ability to engage with a palliative care team (9). Alternative methods of physician engagement such as increased utilization of telehealth platforms have helped bridge geographical barriers creating increased access to palliative care services for those in underserved populations. Many studies show that using telehealth in these settings improves quality of life and decreases symptom burden with high levels of patient satisfaction (10, 11). But perhaps the most notable challenge is due to the limited resource of specialty palliative care with a significant supply and demand disparity. While most NCCN member institutions have outpatient palliative care services, a vast majority have reported inadequate staffing to meet the level of need (12). This effect is compounded further at many non-academic community sites where they not only experience staffing shortages but often limited institutional resources for palliative care program development due to inadequate funding (12). Research shows that community-based oncologists provide a majority of cancer care throughout the country making this of particular concern (13). Despite a stark increase in Accreditation Council for Graduate Medical Education (ACGME) accredited hospice and palliative medicine fellowship positions over the past couple decades, the number of annual graduates will still need to nearly double to provide adequate staffing of United States hospital and community-based palliative care programs in the near future (14, 15). While expanding fellowship positions along with improving reimbursement models would lead to increased supply, creating a precision-based framework for palliative care referral offsetting demand is also of importance. Many advanced/metastatic solid tumor malignancies have widely variable prognoses and degree of symptom burden both between and among primary tumor sites (9). This is due to both recent therapeutic advancements and biologic variability which is highlighted nicely within the scope of metastatic NSCLC. Therefore, blanket recommendations for early palliative care integration for all patients should be refined to ensure palliative care services are offered and available to those patients who will derive the greatest benefit.

## A shift in the NSCLC treatment paradigm

At the time of enrollment and publication of the landmark Temel trial, the therapeutic scope of metastatic NSCLC was starkly limited compared to present day. Then, the backbone of first line treatment for nearly all patients included platinum-doublet chemotherapy. with the addition of maintenance chemotherapy just becoming standard (16). While a limited number of tyrosine kinase inhibitors (TKIs) were starting to gain Food and Drug Administration (FDA) approval in the second line setting, gefitinib in 2003 and erlotinib in 2004 both targeting *EGFR* alterations, the routine use of biomarker testing to guide treatment decisions was yet to become part of guideline recommendations (17, 18). Further, the rise of immunotherapy was still several years away. Median overall survival was estimated around 11 months for patients with metastatic NSCLC diagnosed in this treatment era (19).

Over the past 15 years, treatment options for NSCLC have multiplied at an unprecedented pace through the identification of targetable genomic alterations and the parallel implementation of immunotherapy. Since the approval of front line crizotinib for *ALK*-rearranged metastatic NSCLC in 2011, there are now 39 first line therapy options for a wide range of molecular targets with a similar number of agents available in the subsequent line setting (20) These new treatment modalities have offered significant improvements in survival for select patients. For instance, osimertinib, the most frequently used TKI targeting common *EGFR* mutations, showcased a median overall survival of 38.6 months in the FLAURA trial (21). Similarly, lorlatinib, a commonly used TKI targeting *ALK*-rearrangements, yielded a seven-year progression free survival of 55%, with median overall survival not reached in the CROWN trial (22). These numbers are staggering compared to the expected survival from cytotoxic chemotherapy. Approximately 35% of all NSCLCs will harbor an actionable genomic alteration with the highest prevalence among adenocarcinomas (23). While single gene assays were initially used to identify oncogenic drivers, by 2018 broad molecular profiling with next generation sequencing was widely recommended. Developing alongside targeted therapy was immunotherapy. In 2016 the immune checkpoint inhibitor pembrolizumab emerged as a promising therapeutic addition to the frontline treatment landscape specifically for patients without *EGFR* or *ALK* alterations. With its first indication as monotherapy and later in combination with chemotherapy, it paved the way for various other immunotherapy approvals over the years. In the KEYNOTE-024 trial, pembrolizumab demonstrated a median overall survival improvement by nearly 16 months compared to chemotherapy among patients with PD-L1 ≥ 50% (24). Although less drastic, survival improvements were also seen when used as monotherapy for those with PD-L1 ≥ 1% and in combination with chemotherapy regardless of PD-L1 status (25, 26). In this post targeted therapy and immunotherapy setting, median overall survival for all patients with metastatic NSCLC has improved to approximately 18 months (19). Looking to the future, new and emerging therapeutic drug classes including bispecific antibodies and antibody-drug conjugates will continue to transform the therapeutic landscape altering prognosis and side effect profile for patients with this disease.

What was once considered a relatively homogeneous disease is now recognized as being highly heterogeneous which has important implications on prognosis. Depending on a multitude of factors, anticipated survival at the time of a metastatic diagnosis may range from months to several years. While the presence of a targetable driver mutation is often encouraging, all are not created equal. For instance, a survival advantage is not recognized among those harboring a mutation in *KRAS* compared to wildtype tumors (27). Additionally, non-targetable co-mutations like *STK11*and *KEAP1* can further modulate prognosis, often in an unfavorable manner (28, 29). In the immunotherapy realm, the degree of PD-L1 expression and tumor mutational burden can dictate response to checkpoint inhibition (30, 31). Not only are molecular identifiers important, but clinical features are also telling. The degree and location of metastatic disease along with individual performance status are both important independent prognostic factors (32, 33). With increasing complexity and nuance involved in the diagnosis, treatment and prognostication of patients with metastatic NSCLC, we find ourselves in a new era of precision medicine. A similar approach must be taken toward the integration of palliative care to appropriately meet our patient's unique individual needs.

## Utilizing triggers to promote precision palliative care

The concept of developing triggers to prompt engagement is a useful strategy to facilitate precision palliative care integration. However, successful implementation of this will require consensus referral criteria based on available resources along with an infrastructure allowing for consistent screening and prompt triage to appropriate services (34).

### Acute inpatient triggers

Several studies have analyzed triggers for palliative care consultation in the inpatient setting for those with cancer. In one study, oncologic hospital admissions were actively screened to identify patients with metastatic cancer admitted with uncontrolled symptoms. Lung cancer comprised 23% of the patient cohort. Identification of such patients prompted contact between a palliative medicine physician and the medical oncologist to discuss need for palliative care consultation. This intervention led to a significant increase in specialty palliative care consultation with more frequent goals of care discussions, improved pain and spiritual needs assessment, along with increased hospice referrals, decreased length of hospital stay, and decreased 30-day readmission rates (35). Another method to identify patients in need of acute palliative care services is to utilize tools that may predict increased risk of mortality such as the Supportive and Palliative Care Indicator Tool and Rothman Index. One study identified both as useful tools in predicting poor prognosis and need for early palliative care referral for patients with advanced cancer in a cohort predominantly comprised of lung and gastrointestinal malignancies (36).

### Patient reported outcome triggers

Utilizing patient reported outcomes is an effective way to promote precision-based palliative care in the outpatient setting for those showcasing high symptom burden or distress. Some of the validated tools for measuring patient reported outcomes include the Integrated Palliative Outcome Scale, the National Comprehensive Cancer Network Distress Thermometer and ESAS. Regular use of these tools with integration into the electronic medical record can allow for easy identification of symptom trajectory and cutoff thresholds. This can help direct the need for specific interventions such psychosocial or spiritual support versus symptom management. The former may benefit most from psychology, social work or chaplaincy services while the latter may require physician-led care. Effectively utilizing all members of the palliative care team can improve overall capacity by reserving provider visits for those with uncontrolled symptoms (34). Studies show that patients with advanced cancer who possess a moderate to severe symptom burden based on a tool such as ESAS will derive benefit from a focused palliative care intervention and in fact are at risk of symptom progression in its absence (37, 38). Patients exhibiting only mild baseline symptoms will often remain stable regardless of whether palliative care is provided (37, 38). This suggests that patient reported outcomes can be effectively utilized to guide referrals.

Specific to the lung cancer population, Temel and colleagues evaluated a more patient-centered palliative care approach via a stepped model after recognizing several limitations to the broad implementation of early palliative care following their initial work in 2010. In this noninferiority trial, patients with advanced lung cancer were randomized to receive either standard early palliative care with monthly visits or a stepped approach with an initial evaluation within four weeks of enrollment and subsequent visits only timed at periods of significant transition (i.e., disease progression, toxicity, treatment discontinuation or hospitalization). Patients completed FACT – Lung (L) assessments for quality-of-life analysis every six weeks. When scores declined by 10 or more points, patients in the stepped approach arm were moved to monthly visits mirroring the early palliative care arm for the remainder of the study (39). They found that this method of palliative care delivery was noninferior for patient-reported quality of life based on the FACT-L assessment compared to standard early palliative care (39). In terms of secondary outcomes, patients receiving stepped palliative care had significantly fewer palliative care visits but a similar proportion in each group engaged in discussions surrounding end of life preferences. One area of potential concern was a significantly shorter hospice duration for the stepped palliative care cohort compared to those receiving standard early palliative care (adjusted mean 19.5 vs. 34.6 days) indicating that more frequent engagement can improve this transition (39). However, this trial provides an important alternative to the time intensive approach of current recommendations and highlights the benefit of adjusting to individual patient needs.

### Disease related triggers

In the absence of patient reported outcome triggers (i.e., high symptom burden), certain disease related factors could also act as a trigger to expedite palliative care involvement for patients with anticipated poor prognosis. Overall, this approach is largely hypothesis driven and has yet to be validated through prospective studies. However, it is imperative that we continue to use our developing knowledge of disease biology as an additional tool to help guide decision making throughout the disease continuum. Some older consensus reports have recommended palliative care integration following progression on second line systemic therapy (40). However, in the metastatic NSCLC setting this may require a more nuanced approach. For instance, patients without actionable genomic alterations and negative PD-L1 expression may benefit from early palliative care integration upfront for a historically poor prognosis as outlined in the 2010 Temel trial. Predicting long term response to immunotherapy remains a challenge for those with PD-L1 positive tumors, so palliative care integration may be best suited for those following progression on frontline immunotherapy. Some patients treated with targeted therapy for genomically driven tumors may expect years of disease control allowing for a delay in palliative care referral until they have exhausted targeted therapy options (41). However, careful attention to targetable *KRAS* and *MET* exon 14 skipping mutations should be considered as these alterations have been associated with poor prognosis despite their targetable nature (42). Further, recognition of non-targetable *SMARCA4*, *STK11* and *KEAP1* mutations known for their aggressive phenotype and sometimes treatment resistant nature may help prompt early palliative care involvement for this patient cohort (28, 43). Again, studies in this space are lacking and future prospective work will be needed to confirm optimal timing of palliative care integration based on a tumor's genomic profile in combination with previous systemic therapy approaches. Hospitalizations may also act as a surrogate for limited life expectancy at later stages. One study found that the first inpatient admission with metastatic NSCLC acted as a transition point indicating poor prognosis with a median survival of only 3.8 months (44). The authors urged palliative care referral at this time if not previously in place to improve end of life outcomes (44). Additional factors including malignant spinal cord compression, brain metastases and leptomeningeal disease are all part of consensus guidelines for palliative care referral and often remain appropriate even in the precision medicine era of therapeutics (40).

## Conclusions

Palliative care integration remains an important component of comprehensive oncologic care. However, current recommendations calling for early palliative care integration for all patients with advanced solid tumor malignancies is an unsustainable practice that has not been successfully implemented on a large scale. Precision palliative care is necessary to optimize patient outcomes while as not to overwhelm a specialty in high demand. Overall, palliative care referral should be guided by symptomatic need and prognosis. To prompt more urgent palliative care assessment in the acute inpatient setting we suggest adoption and consistent utilization of validated tools such as the Supportive and Palliative Care Indicators Tool and Rothman Index to identify those with unmet palliative care needs along with those who are at high risk of clinical deterioration. In the outpatient setting, research shows that those appropriate for referral based on symptom burden can be successfully identified through monitoring of patient reported outcomes over time via tools like ESAS. While this requires mutual buy-in from both patients and the broader healthcare system, prospective studies find this to be a feasible and effective method to prompt precision palliative care referral. In the current era, identifying those with limited prognosis in the absence of clinical deterioration is more difficult, but our growing knowledge of disease biology could help bridge this gap. Future studies assessing timing of palliative care referral based on genomic alterations and other tumor biomarkers in metastatic NSCLC are warranted. For example, those with favorable alterations like *ALK* rearrangements or *EGFR* L858R mutations may allow for later palliative care integration only after targeted therapy options have been exhausted whereas those with a *KRAS* G12C mutation may benefit from early palliative care at disease onset or after progression on first line therapy. This could be further modulated by the presence of adverse co-mutations. While this is an area yet to be prospectively analyzed, it represents an innovative opportunity for enhanced precision-based care. By aligning palliative care integration with the same precision medicine focus that guides current oncologic treatment, we can move toward an individualized, sustainable, and clinically meaningful model of supportive care for our patients and health system at large.

## References

[1] National Comprehensive Cancer Network. NCCN Guidelines Version 1.2026 Palliative Care (2026). Available online at: https://www.nccn.org/professionals/physician_gls/pdf/palliative.pdf (Accessed July 6, 2026).

[2] Kelley AS Meier DE. Palliative care–a shifting paradigm. N Engl J Med. (2010) 363(8):781–2. 10.1056/NEJMe1004139 20818881

[3] Petrillo LA Jones KF El-Jawahri A Sanders J Greer JA Temel JS. Why and how to integrate early palliative care into cutting-edge personalized cancer care. Am Soc Clin Oncol Educ Book. (2024) 44(3):e100038. 10.1200/EDBK_100038 38815187

[4] Temel JS Greer JA Muzikansky A Gallagher ER Admane S Jackson VA. Early palliative care for patients with metastatic non-small-cell lung cancer. N Engl J Med. (2010) 363(8):733–42. 10.1056/NEJMoa1000678 20818875

[5] Bakitas MA Tosteson TD Li Z Lyons KD Hull JG Li Z. Early versus delayed initiation of concurrent palliative oncology care: patient outcomes in the ENABLE III randomized controlled trial. J Clin Oncol. (2015) 33(13):1438–45. 10.1200/JCO.2014.58.6362 25800768 PMC4404422

[6] Kang EK Kang JH Koh S-J Kim YJ Seo S Kim JH. Early integrated palliative care in patients with advanced cancer: a randomized clinical trial. JAMA Netw. Open. (2024) 7(8):e2426304. 10.1001/jamanetworkopen.2024.26304 39115845 PMC11310828

[7] Haun MW Estel S Rücker G Friederich H-C Villalobos M Thomas M. Early palliative care for adults with advanced cancer. Cochrane Database Syst Rev. (2017) 6(6):CD011129. 10.1002/14651858.CD011129.pub2 28603881 PMC6481832

[8] Huo B Song Y Chang L Tan B. Effects of early palliative care on patients with incurable cancer: a meta-analysis and systematic review. Eur J Cancer Care (Engl). (2022) 31(6):e13620. 10.1111/ecc.13620 35612356

[9] Mitchell C Chiec L. Integration of early palliative care in personalized cancer care. Advances in Oncology. (2025) 5(1):65–71. 10.1016/j.yao.2024.12.002

[10] Kawashima A Noguchi T Furukawa T Unesoko A Togashi S Sato K. Effectiveness of and implementation requirements for telehealth in palliative care patients with advanced cancer: a systematic review and meta-analysis. Palliat Med. (2026) 40(5):588–607. 10.1177/02692163251403395 41467572 PMC13136543

[11] Baksh A Martin A Pacheco S. Necessity is the mother of implementation: patient satisfaction with telemedicine for palliative care during the COVID-19 pandemic. J Pain Symptom Manage. (2022) 63(5):855–6. 10.1016/j.jpainsymman.2022.02.037

[12] Kayastha N LeBlanc TW. Why are we failing to do what works? Musings on outpatient palliative care integration in cancer care. JCO Oncol Pract. (2022) 18(4):255–7. 10.1200/OP.21.00794 34936378

[13] Nguyen CA Beaulieu ND Wright AA Cutler DM Keating NL Landrum MB. Organization of cancer specialists in US physician practices and health systems. J Clin Oncol. (2023) 41(26):4226–35. 10.1200/JCO.23.00626 37379501 PMC10852402

[14] Lupu D Quigley L Mehfoud N Salsberg ES. The growing demand for hospice and palliative medicine physicians: will the supply keep up? J Pain Symptom Manage. (2018) 55(4):1216–23. 10.1016/j.jpainsymman.2018.01.011 29410071

[15] Dingfield L. Addressing a Workforce Crisis: Innovations in Training for HPM Specialists (2020). Available online at: https://www.capc.org/blog/palliative-pulse-the-palliative-pulse-february-2019-addressing-a-workforce-crisis-innovation-training-for-hpm-specialists/ (Accessed July 30, 2026).

[16] Mirshahidi HR Hsueh CT. Updates in non-small cell lung cancer–insights from the 2009 45th annual meeting of the American society of clinical oncology. J Hematol Oncol. (2010) 3:18. 10.1186/1756-8722-3-18 20433767 PMC2876054

[17] Kazandjian D Blumenthal GM Yuan W He K Keegan P Pazdur R. FDA Approval of gefitinib for the treatment of patients with metastatic EGFR mutation–positive non–small cell lung cancer. Clin Cancer Res. (2016) 22(6):1307–12. 10.1158/1078-0432.CCR-15-2266 26980062

[18] Johnson JR Cohen M Sridhara R Chen Y-F Williams GM Duan J. Approval summary for erlotinib for treatment of patients with locally advanced or metastatic non–small cell lung cancer after failure of at least one prior chemotherapy regimen. Clin Cancer Res. (2005) 11(18):6414–21. 10.1158/1078-0432.CCR-05-0790 16166415

[19] Basse C Carton M Milder M Geiss R Du Rusquec P Daniel C. Real-World survival impact of new treatment strategies for lung cancer: a 2000-2020 French cohort. Cancers (Basel). (2024) 16(15):2768. 10.3390/cancers16152768 39123495 PMC11312246

[20] National Comprehensive Cancer Network. NCCN Guidelines Non-Small Cell Lung Cancer Version 6.2026 (2026). Available online at: https://www.nccn.org/professionals/physician_gls/pdf/nscl.pdf (Accessed July 31, 2026).

[21] Ramalingam SS Vansteenkiste J Planchard D Cho BC Gray JE Ohe Y. Overall survival with osimertinib in untreated, EGFR-mutated advanced NSCLC. N Engl J Med. (2019) 382(1):41–50. 10.1056/NEJMoa1913662 31751012

[22] Peters S Camidge DR Shaw AT Gadgeel S Ahn JS Kim D-W. Alectinib versus crizotinib in untreated ALK-positive non–small-cell lung cancer. N Engl J Med. (2017) 377(9):829–38. 10.1056/NEJMoa1704795 28586279

[23] Russo A Javey M Huang RSP Yaung SJ Kaplan B Hiemenz MC. Molecular profiling across 80,000 patients with lung cancer. J Thorac Oncol. (2026) 21:103695. 10.1016/j.jtho.2026.103695 41903702

[24] Reck M Rodríguez-Abreu D Robinson AG Hui R Csőszi T Fülöp A. Pembrolizumab versus chemotherapy for PD-L1–positive non–small-cell lung cancer. N Engl J Med. (2016) 375(19):1823–33. 10.1056/NEJMoa1606774 27718847

[25] Gandhi L Rodríguez-Abreu D Gadgeel S Esteban E Felip E De Angelis F. Pembrolizumab plus chemotherapy in metastatic non-small-cell lung cancer. N Engl J Med. (2018) 378(22):2078–92. 10.1056/NEJMoa1801005 29658856

[26] de Castro G Kudaba I Wu Y-L Lopes G Kowalski DM Turna HZ. Five-Year outcomes with pembrolizumab versus chemotherapy as first-line therapy in patients with non–small-cell lung cancer and programmed death ligand-1 tumor proportion score ≥ 1% in the KEYNOTE-042 study. J Clin Oncol. (2023) 41(11):1986–91. 10.1200/JCO.21.02885 36306479 PMC10082298

[27] Carbone DP Hirsch FR Osarogiagbon RU Nishimura KK Tsao MS Travis WD. The international association for the study of lung cancer staging project: the impact of common molecular alterations on overall survival in NSCLC in initial analyses of the IASLC ninth edition staging database. J Thorac Oncol. (2025) 20(10):1423–40. 10.1016/j.jtho.2025.06.025 40920144

[28] De Giglio A De Biase D Favorito V Maloberti T Di Federico A Zacchini F. STK11 Mutations correlate with poor prognosis for advanced NSCLC treated with first-line immunotherapy or chemo-immunotherapy according to KRAS, TP53, KEAP1, and SMARCA4 status. Lung Cancer. (2025) 199:108058. 10.1016/j.lungcan.2024.108058 39709652

[29] Papillon-Cavanagh S Doshi P Dobrin R Szustakowski J Walsh AM. STK11 And KEAP1 mutations as prognostic biomarkers in an observational real-world lung adenocarcinoma cohort. ESMO Open. (2020) 5(2):e000706. 10.1136/esmoopen-2020-000706 32312757 PMC7199918

[30] Ricciuti B Wang X Alessi JV Rizvi H Mahadevan NR Li YY. Association of high tumor mutation burden in non–small cell lung cancers with increased immune infiltration and improved clinical outcomes of PD-L1 blockade across PD-L1 expression levels. JAMA Oncol. (2022) 8(8):1160–8. 10.1001/jamaoncol.2022.1981 35708671 PMC9204620

[31] Tan J Yang S-C Dinan MA Chiang AC Gross CP Wang S-Y. Biomarker-Specific survival and medication cost for patients with non–small cell lung cancer. JAMA Netw. Open. (2025) 8(6):e2514519. 10.1001/jamanetworkopen.2025.14519 40493365 PMC12152704

[32] Metzenmacher M Griesinger F Hummel H-D Elender C Schäfer H De Wit M. Prognostic factors in nonsmall cell lung cancer: insights from the German CRISP registry. Eur Respir J. (2023) 61(2):2201336. 10.1183/13993003.01336-2022 36180086 PMC9892864

[33] Kawaguchi T Takada M Kubo A Matsumura A Fukai S Tamura A. Performance Status and smoking Status are independent favorable prognostic factors for survival in non-small cell lung cancer: a comprehensive analysis of 26,957 patients with NSCLC. J Thorac Oncol. (2010) 5(5):620–30. 10.1097/JTO.0b013e3181d2dcd9 20354456

[34] Sedhom R Shulman LN Parikh RB. Precision palliative care as a pragmatic solution for a care delivery problem. J Clin Oncol. (2023) 41(16):2888–92. 10.1200/JCO.22.02532 37084327 PMC10414742

[35] Hanson LC Collichio F Bernard SA Wood WA Milowsky M Burgess E. Integrating palliative and oncology care for patients with advanced cancer: a quality improvement intervention. J Palliat Med. (2017) 20(12):1366–71. 10.1089/jpm.2017.0100 28737996 PMC5749575

[36] Chan AS Rout A ’Adamo CRD Lev I Yu A Miller K. Palliative referrals in advanced cancer patients: utilizing the supportive and palliative care indicators tool and rothman Index. Am J Hosp Palliat Care. (2022) 39(2):164–8. 10.1177/10499091211017873 34002623

[37] Lee G Kim HS Lee SW Park YR Kim EH Lee B. Pre-screening of patient-reported symptoms using the Edmonton symptom assessment system in outpatient palliative cancer care. Eur J Cancer Care (Engl). (2020) 29(6):e13305. 10.1111/ecc.13305 33016473

[38] Zimmermann C Pope A Hannon B Krzyzanowska MK Rodin G Li M. Phase II trial of symptom screening with targeted early palliative care for patients with advanced cancer. J Natl Compr Canc Netw. (2021) 20(4):361–70.e3. 10.6004/jnccn.2020.7803 34492632

[39] Temel JS Jackson VA El-Jawahri A Rinaldi SP Petrillo LA Kumar P. Stepped palliative care for patients with advanced lung cancer: a randomized clinical trial. Jama. (2024) 332(6):471–81. 10.1001/jama.2024.10398 38824442 PMC11145511

[40] Hui D Mori M Watanabe SM Caraceni A Strasser F Saarto T. Referral criteria for outpatient specialty palliative cancer care: an international consensus. Lancet Oncol. (2016) 17(12):e552–e9. 10.1016/S1470-2045(16)30577-0 27924753

[41] Sampetrean A Aldea M Mateus C. Precision medicine's New frontier: integrating palliative care at the right time. ESMO Open. (2023) 8(5):101632. 10.1016/j.esmoop.2023.101632 37757666 PMC10534217

[42] Tóth LJ Mokánszki A Méhes G. The rapidly changing field of predictive biomarkers of non-small cell lung cancer. Pathology and Oncology Research. (2024) 30:1611733. 10.3389/pore.2024.1611733 38953007 PMC11215025

[43] Schoenfeld AJ Bandlamudi C Lavery JA Montecalvo J Namakydoust A Rizvi H. The genomic landscape of SMARCA4 alterations and associations with outcomes in patients with lung cancer. Clin Cancer Res. (2020) 26(21):5701–8. 10.1158/1078-0432.CCR-20-1825 32709715 PMC7641983

[44] Collins A Sundararajan V Burchell J Millar J McLachlan S-A Krishnasamy M. Transition points for the routine integration of palliative care in patients with advanced cancer. J Pain Symptom Manage. (2018) 56(2):185–94. 10.1016/j.jpainsymman.2018.03.022 29608934

